# Supplementary material for: Copy number signature analysis tool and its application in prostate cancer reveals distinct mutational processes and clinical outcomes
Source: PLoS Genet. 2021 May 4;17(5):e1009557. doi: 10.1371/journal.pgen.1009557 (PMC8121287; doi:10.1371/journal.pgen.1009557)
Supplement: Attachment — Submitted filename: Response to Reviewer.docx [file pgen.1009557.s019.docx]

**Manuscript # PGENETICS-D-20-01922**

**Responses to Reviewers' comments**

**Reviewer #1:**

*The authors present sigminer, a tool able to identify signatures of copy number alterations. They use it to study copy number and single base substitution signatures in prostate cancer. They identify five copy number signatures (CNA signatures) and three single base substitution signatures (SBS signatures) in 1003 samples. They describe the association between CN-Sig 2 (the second copy number signature) and prostate cancer metastasis and a significant association with poor overall survival. CN-Sig 5 is associated with improved overall survival. In addition, they cannot find a clear association between most clinical parameters in prostate cancer and the three SBS signatures. They conclude that CNA signatures are more useful for patient stratification compared to SBS signatures in prostate cancer.
I think that sigminer can be useful to easily analyse other cancer types.

Comments:
1-The authors compare their method to a previously published method (Macintyre et al.). What is the relation between the five CNA signatures in this study and the seven CNA signatures described in Macintyre et al. in ovarian carcinoma? Are some of them similar? Considering fig S1A, they seem quite different.*

**Response:** Thanks to the reviewer for this comment. The signatures reported in this study and those of Macintyre et al. are based on different method, and different CNA component or features are included in these two methods. Cosine similarity analysis cannot be performed between these two sets of signatures. The CNA signature of Fig S1A is for demonstration. The similarity between these two sets of signatures cannot be performed, because these two sets of signatures are composed of different signature components.

*2-One of the novelty of sigminer is that it supports single sample analysis. However, this functionality is particularly interesting when we have a set of reference CNA signatures. Are the 5 CNA signatures identified in prostate cancer present also in other cancer types? Can these 5 CNA signatures considered reference signatures?*

**Response:** We agree with the reviewer that reference signature need to be provided for single sample analysis. Some signatures identified in prostate cancer can also be found in other cancer types, for example CN-Sig3 reflects whole-genome duplication, and this signature is commonly seen in other cancer types. Some of the signatures identified in prostate cancer could be cancer type specific. The five CNA signatures reported in this study can be reference signatures for prostate cancer. We agree with the reviewer, sigminer can be applied to other cancer types, **S1 Fig** is an application of this method in ovarian cancer.

**Reviewer #2:**

*Manuscript by Wang et al. describe a new method/algorithm for copy number signature analysis in colorectal cancer. As the authors suggest there is definitely a need for such an algorithm. The manuscript as written needs major revision. The language throughout needs extensive review and revision for clarity. too much mix of passive and direct statements. too much repetition. There needs to be sanity checks on the initial calculation of copy numbers and mutations.

Why not proceed with matching copy numbers from both facets and Sequenza? Why not follow something similar to the TCGA protocol which involves multiple callers and careful filtering to remove false positives?. what was the overlap with the TCGA data?*

**Response:** Thanks to the reviewer for this comment. As suggested, we now carefully checked the TCGA protocol, and the CNA profiles generated have been carefully filtered with multiple methods. We compared the performance between Sequenza and FACETS. For most samples, Sequenza and FACETS method report similar results (**Rebuttal Figure 1**). However, we observed many long regions (>10Mb) deep deletion in about 20 samples from FACETS data (**Rebuttal Figure 2**), but not Sequenza data. It is unlikely to find so many deep deletions in a tumor, thus in original manuscript Sequenza data is used.

**Rebuttal Figure 1. Comparison of copy number profiles generated from FACETS and Sequenza.** Copy number profile similarity density plot comparing two methods. Cosine similarity was calculated based on segment-wise copy number in each tumor.

**Rebuttal Figure 2**. A copy number profile generated by FACETS.

The CNA profiles generated in this study are derived from WES data, while the CNA data of TCGA datasets are from SNP array data of the same patient. We compared our CNA profile data with TCGA CNA data downloaded from GDC portal, they are very similar (**Rebuttal Figure 3**), demonstrating the reliability of our data processing steps.

**Rebuttal figure 3. Comparison of copy number profiles downloaded from TCGA GDC portal (based on SNP array) with our in-house CNA calling data (from WES using the same TCGA samples) (Dataset: Phs000178, n=486).** (**A**) Distribution of copy number profile cosine similarity between two platforms (SNP/WES). Cosine similarity was calculated based on segment-wise copy number in each tumor. (**B**) Copy number profile of 3 tumors (with stable genome, many deletions and many amplifications, respectively) as comparison examples. The top panel indicates data downloaded from TCGA and the bottom panel indicates data from this study using WES of the same patient.

*Did you check the tcga mutation calls with your own calls? What was the overlap? Why did you only use mutect2? Strelka, varscan2 etc?*

**Response:** As suggested, we compared the results from our own mutation calling with TCGA mutation calls using TCGA prostate cancer dataset (Dataset: Phs000178), results indicate that most of our mutation calls overlap with TCGA results (**Rebuttal Figure 4A**). We further compare the mutation calls with 4 common variant calling methods, majority of mutations are overlapping in all methods (**Rebuttal Figure 4B**).

**Rebuttal figure 4.** Variant calling comparison using TCGA prostate cancer dataset. (**A**) Comparison between our mutation calling results with TCGA results. (**B**) Variants calling comparison using 4 commonly used methods.

*Page 21: "Next, unlike previous study[15], which applied mixture modeling to separate the 6 copy number features’ distributions into mixtures of Poisson or Gaussian distributions, we directly classified 8 copy number features’ distributions into 80 components according to the comprehensive consideration of value range, abundance and biological significance (Figure S4e).”

What is the rationale behind this? What is the biological significance of the features? Explain. There is comparison of the two methods in figs1 but this is not clearly explained.*

**Response:** Thanks to the reviewer for this comment. The key difference between our method and Macintyre et al. is the definition of CNA feature and component. Our method apply a predefined CNA component, each component has a clear biological meaning, for example: “CN[2]” indicate the absolute copy number of the DNA segment is 2. In total we have 80 CNA components. However, the biological meaning of the CNA component in Macintyre et al. method is not clear and predefined. For each different dataset, a different CNA component set will be used. This is problematic especially if the investigator want to compare the signature generated with different dataset using cosine similarity analysis (See additional **S1 Fig**). Due to this problem Macintyre et al. method cannot be applied in single sample CNA signature analysis.

*pg 23: “SBS signature profile was normalized within each signature (i.e. by row). We performed row normalization within each copy number feature, so a component value can be compared to another component value of the same copy number feature within each signature. In summary, we performed row normalization for SBS signature profile and row normalization within each feature for copy number signature.”

Repeating the same thing. Simplify.*

**Response:** Thanks to the reviewer for this comment. As suggested, the repetitive words have been deleted.

*Why neural network? How does this signature assignment compare to nonlinear least squares or iterative fitting/complete enumeration?*

**Response:** Thanks to the reviewer for this comment. The purpose of this neural network is to classify cancer samples based on CNA signature, it is not for CNA signature assignment.

*Page 9
"Macintyre et al employed a mixture modeling based method for copy number component extraction, and this method cannot be directly applied to other cancer types”

Why not? I see no reason that these methods cannot be applied to other cancers than ovarian. This is not clear enough to reasoning to justify the methods used.*

**Response:** We agree with the reviewer that Macintyre et al. method can be applied to other cancer types in principle. However for analysis in different cancer types, the components of the signatures will be different with Macintyre et al. method. See our additional analysis (**S1 Fig**). This prohibit the traditional signature comparisons with cosine similarity analysis (**S1 Fig**). The related information has now been included in the revised manuscript, and the text has been revised accordingly.

*Page 9. 143-145. This is almost word to word repeat of the method. Again, there is no explanation as to what consists of biological significance.*

**Response:** Thanks to the reviewer for this comment. As suggested, the text has been revised. New text has been added to illustrate the biology of our predefined CNA component (Line 144-147).

*Page 10. para starting with line 155. very unclear. it turns out you actually compared to previous work. Clearly state that. at the same time all the relevant details are buried in the S1 fig legend. Move those details to main text. in addition, explain what is the clear and fixed biological meaning of these 80 features. Why did you only compare your signature 1 with the previous method in Fig S1? I thought there were 3 signatures based on your method. Since both methods used similar features an in depth discussion of the similarities and differences is clearly warranted.*

**Response:** As suggested, this paragraph has been extensively edited. The key difference between our method and Macintyre et al. method now has been clearly stated. In addition, we included new data to illustrate the difference between our method and Macintyre et al. method using TCGA ovarian cancer dataset (**S1 Fig**).

*Page 10 last para. is another repeat statement. why? I don't need to read in every section you created a user friendly bioinformatic tool.*

**Response:** As suggested, this paragraph has been modified, and the repetitive statement has been deleted.

**Reviewer #3:**

*In this study, Wang et al. create a bioinformatic tool called sigminer to extract copy number signatures from tumor sequencing data. They apply this tool to a WES prostate cancer dataset containing both primary and metastatic samples and make observations about underlying mutational processes and association with clinical outcomes.*

*In general, copy number signatures may represent a valuable tool alongside more established mutational signatures as well as recently described structural variant signatures (Glodzik et al. Nat Genetics 2017, Nik-Zainal et al., Nature 2016), both in terms of gleaning insight into underlying mutational processes of cancer and as clinical biomarkers. However, the major limitations of this study are: 1) lack of novelty and demonstration of how this approach represents a conceptual advance over the study by Macintyre et al. Nat Genetics 2018; 2) lack of clear evidence that the CN signatures authors have derived will lead to meaningful biological insights related to prostate cancer.*

*Addressing the points below would substantially strengthen the manuscript.*

*A. Because Macintyre et al have already introduced the concept of copy number signatures, it is important for the authors to compare their method and the Macintyre et al. method head-to-head in the same datasets. The authors make some statements to suggest that their method has some advantages but this is not clearly demonstrated on actual data and it is unclear if these differences are substantive.*

**Response:** Thanks to the reviewer for this comment. The key difference between our method and Macintyre et al. is the definition of CNA feature and component. Our method apply a predefined CNA component, each component has a clear biological meaning, for example: “CN[2]” indicate the absolute copy number of the DNA segment is 2. In total we have 80 CNA components. However, the biological meaning of the CNA component in Macintyre et al. method is not clear and predefined. For each different dataset or cancer type, a different CNA component set will be used. This is problematic especially if the investigator want to compare the signature generated with different dataset using cosine similarity analysis.

As suggested, we now performed CNA signature analysis in ovarian cancer with our method and Macintyre et al. method (**S1 Fig**). To facilitate the understanding of the difference, we applied these two copy number signature extraction methods to a subset TCGA ovary cancer cohort with different sample size (**S1 Fig A-C**). Four signatures are extracted independently from datasets with 50 samples, 20 samples and 10 samples, respectively. We can observe that the component number is consistent for three datasets with our method but not Macintyre et al. method. The unequal components (unequal component number and meaning) make the cosine similarity analysis impractical to be applied using Macintyre et al. method. Our method could report stable and consistent result, the cosine similarity analysis shows that several copy number signatures can be stably identified with different sample size using our method (**S1 Fig D**).

*B. The authors should explain how their cohort of prostate cancer samples were chosen. Based on the references cited (#10, #20-24), this represents quite a heterogeneous set of samples both in terms of treatment stage (reference 10 represents CRPC, 24 PDXs that are enzalutamide resistant, 23 represents CRPC that is a mixture of enzalutamide-naïve and resistant, and 22 represents neuroendocrine prostate cancer, and 20-21 are localized prostate cancer). In particular, the use of samples from reference 22 is potentially problematic as neuroendocrine PCa has a very different biology and mutational spectrum than adenocarcinoma and should not be mixed for analysis. Would recommend that the authors use the ~1000 WES samples in the Armenia et al. Nature Genetics 2018 study for a more uniformly curated cohort. The use of this heterogeneous cohort may have significant influence on the clinical correlations obtained in the current version of the manuscript.*

**Response:** Thanks to the reviewer for this comment. Actually our initial prostate cancer genome dataset selection was based on the reference mentioned by the reviewer (Armenia et al. Nature Genetics 2018). The datasets included in Armenia et al. Nature Genetics 2018 study: phs000178, phs000447, phs000909, phs000915, phs000945. The datasets included in our study: phs000178, phs000447, phs000909, phs000915, phs001141. phs000554. Difference between Armenia et al. Nature Genetics 2018 and out study: 1, phs000945 of Armenia et al. cannot be downloaded in our study. 2, We have two additional dataset phs000554、phs001141. The particular reference and dataset “phs000909 [22]” mentioned by the reviewer was also included in Armenia et al. Nature Genetics 2018 study. See the Supplementary Table 1 of Armenia et al. Nature Genetics 2018: (https://www.nature.com/articles/s41588-018-0078-z). We agree with the reviewer that different types of prostate cancer can have different biology and mutational spectrum. This study aims to investigate the diverse mutational processes in prostate cancer, thus we included as many types of prostate cancer as possible. Similar to Armenia *et al*. Nature Genetics 2018 paper, all the raw data are uniformed processed in this study.

*C. Most copy number changes are really a result of underlying structural variants (rearrangements). The authors need to discuss their method in the context of recently described rearrangement signatures (Nik-Zainal et al. Nature 2016, Menghi et al., PNAS, 2016, etc). One could argue that CN signatures and rearrangement signatures report on the same process. In that case, CN signatures could still be useful for certain data types (WES, cfDNA, shallow WGS) where RS derivation is not possible. But then the authors need to show this formally.*

**Response:** We agree with the reviewer that copy number signature and the rearrangement signature reported by Nik-Zainal et al. could have similar underlying mutational processes. As the reviewer pointed out one of key difference between copy number signature and the previously described rearrangement signature is that copy number signature can be obtained with many types of data, such as WGS, WES, SNP array, while the previously described rearrangement signature can only be obtained with WGS data. The related information and citation have now been included in the revised discussion part (Line 417-418).

*D. If the authors want to make claims about significance beyond this being a methods-only paper, signature analysis should be extended to other tumor types and/or validation datasets should be used for findings within a tumor type.*

**Response:** As suggested, we now provide new analysis in ovarian cancer using the bioinformatics tool developed in this study. The performance of this tool has also been compared with existing method (**S1 Fig**).

*E. Points regarding specific signatures:
1) Can the authors comment on why SBS-2 and 3 are so similar?*

**Response:** SBS-2 and SBS-3 are actually not similar. SBS-2 is similar to COSMIC Sig 6, SBS-3 is similar to COSMIC Sig 1. The reference COSMIC signatures are available:

Sig6（https://cancer.sanger.ac.uk/signatures_v2/Signature-6.png）and

Sig1（https://cancer.sanger.ac.uk/signatures_v2/Signature-1.png）

*2) The authors imply that CN-2 is a metastasis-specific biomarker because it is enriched in metastatic prostate cancer samples. But this is really a meaningful predictive finding if the authors can show that this signature is in primary prostate cancer that subsequently more likely to become metastatic.*

**Response:** Our data imply that CN-Sig 2 is associated with prostate cancer metastasis, suggesting CN-Sig 2 can be prostate cancer stratification biomarker. However currently we do not have enough evidence to suggest that CN-Sig 2 can be predictive biomarker for prostate cancer metastasis. For predictive biomarker performance evaluation, we need to collect samples when prostate cancer are at primary and localized stage, and follow the patients for sufficient time to see if metastasis occur or not, and this type of samples are lacking in our datasets.

*3) If the authors plan to make a claim that CN-1 is associated with ecDNA this should be validated, if not experimentally, then at least with computational tools.*

**Response:** Thanks to the reviewer for this comment. Currently we do not have sufficient data to support the association between CN-Sig 1 and ecDNA. In the original manuscript, we make the prediction that “some of them can be caused by the formation of extrachromosomal DNA (ecDNA)”. As suggested, this prediction has been deleted in the revised text.

*4) The fact that CDK12 mutations and other HRD mutations (BRCA1/2) are in the same signature is somewhat concerning. This is not consistent with rearrangement signatures in prostate and breast cancers where BRCA mutations are associated with small span size SVs than CDK12-associated duplications. Moreover, the clinical data from the TRITON study and others suggest that the response rate of CDK12-mutant prostate cancer to PARP inhibitors is virtually nonexistent, as compared with the response rate of BRCA mutant prostate cancer to these agents. So the current data do not suggest a role for CDK12 in homologous recombination repair in prostate cancer.*

**Response:** We agree with the reviewer that *CDK12* mutations and *BRCA* mutations are usually associated with different genome DNA alteration patterns, and *CDK12* is not a major player in homologous recombination repair. The co-occurrence of *CDK12* mutations and *BRCA* mutations in CN-sig 2 could indicate that this signature is a mixture in some aspect, it is not caused simply by HRD, we labeled it as “Tandem duplication” in the original text. And both *BRCA1* mutation and *CDK12* mutation have been reported to be able to cause tandem duplication (Menghi et al. Cancer Cell. 2018 Aug 13;34(2):197-210).

*5) Overall, the use of CN signatures for meaningful risk stratification in prostate cancer and superiority over existing risk stratification schemes has not been clearly shown in this manuscript.*

**Response:** Existing risk stratification factors for prostate cancer include: tumor state, Gleason score, CNA burden. In S12 Fig, we evaluated the performance of these existing stratification schemes. Compared to CNA burden, the activity of some CNA signature do have improved stratification performance (Fig 6). This information has been included in the revised manuscript (Line 375-383).
