# Supplementary material for: Copy number signature analysis tool and its application in prostate cancer reveals distinct mutational processes and clinical outcomes
Source: PLoS Genet. 2021 May 4;17(5):e1009557. doi: 10.1371/journal.pgen.1009557 (PMC8121287; doi:10.1371/journal.pgen.1009557)
Supplement: Attachment — Submitted filename: Response to reviewer R2.docx [file pgen.1009557.s020.docx]

**Manuscript # PGENETICS-D-20-01922R1**

**Responses to Reviewers' comments**

*Reviewer #1: The authors addressed appropriately my two comments by presenting new analysis in S1 Fig.*

*Reviewer #2: The authors have addressed my questions.*

*Reviewer #3: In the revised manuscript, Wang et al. have made several textual clarifications and present new data in Figure S1 in which they perform CNA signature analysis in the TCGA ovarian cancer data using both their method and the Mcintyre et al. method.*

*Overall, while the authors have clarified some issues and sigminer seems like a reasonable new method for CN signature analysis, the manuscript remains largely similar to the first submission and my major reservations are primarily regarding the strength of biological insights that can be drawn at this stage, specifically:*

*1. Fig S1 – the authors run their method and the McIntyre et al. method on the TCGA ovarian cohort and report that there are some numerical differences (number of signatures extracted, number of components etc.). But what does this actually mean biologically?*

**Response:** The major difference between our method and Macintyre et al. method is the definition of copy number features and components. The copy number features and components have clear and predefined biological meaning in our method, while Macintyre et al. method do not. In different dataset, a different set of copy number components will be applied when using Macintyre et al. method. This inconsistence in the definition of copy number component make Macintyre et al. method not applicable in comparing the signatures generated with different dataset or in different cancer types. All these differences have been clearly stated in the revised manuscript.

*Can the authors comment on what underlying genomic processes the McIntyre signature is reporting on versus the authors’ signature and what are the reasons, if any, for the differences?*

**Response:** The underlying mutational processes cannot be directly derived from the copy number signature profiles. Association studies need to be performed to link the potential mutational processes to copy number signatures. As suggested, the copy number signature profiles generated with Macintyre et al. method are shown as below (**Rebuttal Fig** 1):

**Rebuttal Fig 1**. Copy number signatures generated with Macintyre et al. method using prostate cancer datasets collected in this study. The statistic mean (this is not presented in Macintyre et al. 2018 study) is shown on the top for each copy number component to help understanding the statistical distribution of each component, and visually inspecting the similarity between signatures extracted with Macintyre et al. method and our method.

Accurate similarity analysis cannot be performed to compare the differences and similarities between the copy number signature generated with Macintyre et al. method and our method. Based on the visual inspection of the representative copy number profiles, the copy number signatures generated with Macintyre et al. method can be linked to the copy number signatures reported in our study:

| **Our study** | **Macintyre et al** | **Notable shared features** |
| --- | --- | --- |
| CN-Sig 1 | CN-Sig 1 | Segments with high copy number (mostly >8) and small segment size. |
| CN-Sig 2 | CN-Sig 2 | Segments with 3, 4 copy number and relatively small segment size. |
| CN-Sig 3 | CN-Sig 4 | Segments with 1, 2 copy number and  middle segment size. |
| CN-Sig 4 | CN-Sig 3 | Segments with copy number ranges from 4-8 and middle segment size. |
| CN-Sig 5 | CN-Sig 5 | Segments with 2 copy number and largest segment size. |

**Rebuttal Fig 2**. Visual inspection to link the copy number signatures generated with Macintyre et al. method and our method. Five copy number signatures extracted with both methods can be matched with visual inspection largely based on features “CN” (copy number) and “SS” (segment size).

*Which is more faithful to what is known about the biology of the disease?*

**Response:** The signatures of copy number alteration in prostate cancer has not been previously reported. As to the copy number signature, or mutational processed involved for prostate cancer, we do not have known knowledge. Thus we cannot accurately claim which method is better in representing the biology of prostate cancer. However, here we clearly demonstrate that the applicability of our method is much more improved compared to Macintyre et al. method.

*The authors state repeatedly that cosine similarity cannot be performed between the two signatures but likely they can be compared in some other meaningful way(s).*

**Response:** Visual inspection and prediction surely can be performed, however these are not accurate comparisons. Since the values of copy number components are changing with different datasets in Macintyre et al. method. The signatures profiles cannot be accurately compared, and this is an inherent problem of Macintyre et al. method.

*2. Cohorts used for prostate cancer analysis – the authors provided clarification that the samples used are similar to those used in the Armenia study. This clarification is important but the point remains that the Armenia study included a mix of about 2/3 localized prostate cancer and 1/3 metastatic prostate cancers. If these are being treated together for Fig 6, S12 etc the conclusions may not be meaningful. Primary and metastatic prostate cancer are very different biologically and in terms of outcomes and should not be pooled into a single cohort.*

**Response:** Thanks for this point. Since several prostate cancer datasets collected in this study do not contain OS or PFS data. In the original Fig 6B and S12B Fig, PFS analysis were performed with phs000178 dataset (TCGA, 498 primary prostate cancer). In Fig 6A and Fig S12A, OS analysis were performed with phs000178 dataset (TCGA, 498 primary prostate cancer) and phs000554 dataset (48 metastatic prostate cancer). As suggested by the reviewer, we performed OS analysis with only TCGA dataset, the result is shown below (**Rebuttal Fig** 3 and **Rebuttal Fig** 4):

**Rebuttal Fig 3**. Forest plots showing the relative risk of signature activities in overall survival with TCGA primary prostate cancer dataset.

**Rebuttal Fig 4**. Forest plots showing the relative risk of selected genomic and clinical features in overall survival with TCGA primary prostate cancer dataset.

The trends are similar, however the statistical P values have been changed in Rebuttal Fig 3, 4 compared with the original Fig 6A and S12A Fig. Specifically in Rebuttal Fig 3, the P values for CN-sig 2 and CN-sig 5 are increased compared to the original Fig 6A. This difference can be explained by the data presented in our S11 Fig. Since CN-sig 2 is closely associated with metastatic prostate cancer, while CN-sig 5 is closely associated with primary prostate cancer, and there is big difference in the prognosis of primary and metastatic prostate cancer. All these information has now been clearly stated in the revised figure legend and text.

*3. Response to comment C – Understood that the CN analysis method can be run on WES and SNP array data while SV signatures require WGS data. My comment was intended to suggest that authors should/could run both CN and SV signatures on a WGS cohort so that they can comment on which CN/SV signatures are reporting on similar underlying mutational processes.*

**Response:** This is an interesting idea, however we currently do not have enough WGS data for SV analysis in prostate cancer. In addition, the current SV analysis method (proposed in Serena Nik-Zainal et al. Nature 534, 47–54 2016 study) did not include several of the features employed in this study, consequently the SV signature will not capture the detailed copy number information proposed in this study.

*4. CN-Sig2 – the authors state that this signature contains both BRCA and CDK12 mutations as both are associated with tandem duplications. While this may be an explanation for why both mutations are enriched among CN-Sig2, this calls into question the biological significance of the authors’ CN signatures. We already know that BRCA and CDK12 mutations are different with respect to mechanism, the size of tandem duplications they cause, and the therapeutic vulnerabilities they unveil. Can the authors comment on the practical use of a CN signature that lumps these biologically two disparate mutations together?*

**Response:** *BRCA* and *CDK12* mutations are different, and the detailed mutational profiles generated by these two mutations could be different. However both *BRCA* and *CDK12* mutations can produce tandem duplication. Similarly *BRCA1* and *BRCA2* mutations are different, since the protein products encoded by these two genes are involved in different steps of homologous recombination (HR). *BRCA1*- inactive cancers dominantly harbor short (<10 kb) tandem duplications, but *BRCA2*-mutant cancers primarily show deletions (Yi et al. Experimental & Molecular Medicine. 50, 1–11. 2018). However in copy number signature analysis, both *BRCA1* and *BRCA2* mutations are linked to one HR defect signature. With the availability of more data, *BRCA* and *CDK12* mutations related copy number signatures could be separated. However we believe this is not a problem for the current copy number signature analysis.

*5. Fig S12 – It seems to me like almost all the confidence intervals cross 1 except CNA burden which has a HR of 1.04 for OS and 1.05 for PFS, both almost crossing one. Can the authors confirm the following about this analysis?*

*a. How was CNA burden calculated and was it different than Hieronymous et al. elife 2018, which has shown that the HR for CNA burden in prostate cancer is on the order of ~1.4 in prostate cancer? Can the authors explain this discrepancy?*

**Response:** Thanks the reviewer for this point. In Hieronymous et al. eLife 2018 study, the PFS HR mean of CNA burden in TCGA prostate cancer is about 1.25 (Fig 2C of Hieronymous et al. eLife 2018). While in our study, the trends are the same, and the PFS HR mean value is about 1.05. We carefully double checked our data and data processing steps, and can confirm that the HR values reported in our study are correct. Our detailed data processing steps and raw data can be available in the “Data and code availability” section of this manuscript. We also carefully checked the supplementary files of Hieronymous et al. eLife 2018 paper, however we could not obtain the raw data for their Fig 2C. The different data source (SNP for Hieronymous et al. and WES for our study) for calculating CNA burden may be another explanation for this HR difference.

b. Was this HR in Fig S12 calculated on both primary and metastatic samples taken together? OS and PFS need to be assessed for each disease state individually.

**Response:** Since several prostate cancer datasets collected in this study do not contain OS or PFS data. In Fig S12A, OS analysis were performed with phs000178 dataset (TCGA, 498 primary prostate cancer) and phs000554 dataset (48 metastatic prostate cancer). In Fig S12B, PFS analysis were with phs000178 dataset (TCGA, 498 primary prostate cancer). As suggested we also performed OS analysis using only TCGA primary dataset, and the results are shown in **Rebuttal Fig 4**. The results of Rebuttal Fig 4 and our original Fig S12A are similar. This dataset usage information has now been included in the revised figure legend.

c. Was this done as univariate or multivariable analysis?

**Response:** The survival analysis was done in univariate way to individually explore the relationship between variable and patient’s OS/PFS time.
